# Electromyographic Activity of Hand Muscles in a Motor Coordination Game: Effect of Incentive Scheme and Its Relation with Social Capital

**DOI:** 10.1371/journal.pone.0017372

**Published:** 2011-03-25

**Authors:** Roberto Censolo, Laila Craighero, Giovanni Ponti, Leonzio Rizzo, Rosario Canto, Luciano Fadiga

**Affiliations:** 1 University of Ferrara, Ferrara, Italy; 2 The Italian Institute of Technology, Genova, Italy; The University of Western Ontario, Canada

## Abstract

**Background:**

A vast body of social and cognitive psychology studies in humans reports evidence that external rewards, typically monetary ones, undermine intrinsic motivation. These findings challenge the standard selfish-rationality assumption at the core of economic reasoning. In the present work we aimed at investigating whether the different modulation of a given monetary reward automatically and unconsciously affects effort and performance of participants involved in a game devoid of visual and verbal interaction and without any perspective-taking activity.

**Methodology/Principal Findings:**

Twelve pairs of participants were submitted to a simple motor coordination game while recording the electromyographic activity of First Dorsal Interosseus (FDI), the muscle mainly involved in the task. EMG data show a clear effect of alternative rewards strategies on subjects' motor behavior. Moreover, participants' stock of relevant past social experiences, measured by a specifically designed questionnaire, was significantly correlated with EMG activity, showing that only low social capital subjects responded to monetary incentives consistently with a standard rationality prediction.

**Conclusions/Significance:**

Our findings show that the effect of extrinsic motivations on performance may arise outside social contexts involving complex cognitive processes due to conscious perspective-taking activity. More importantly, the peculiar performance of low social capital individuals, in agreement with standard economic reasoning, adds to the knowledge of the circumstances that makes the crowding out/in of intrinsic motivation likely to occur. This may help in improving the prediction and accuracy of economic models and reconcile this puzzling effect of external incentives with economic theory.

## Introduction

The assumption of *Homo Oeconomicus* at the basis of economic reasoning entails the prediction that individuals should respond to incentives, altering costs and benefits associated to available choices, in an manner consistent with a self-regarding behavior. Since the early 70's , however, a large body of empirical research undertaken by social and cognitive psychologists shows that in many social contexts external rewards, typically monetary ones, affect behavior in a direction opposite to that predicted by a standard selfish-rationality argument. This evidence strongly supports the view that *external motivations* often undermine *intrinsic motivations*, which *per se* sustain effort and performance, resulting ineffective or even counterproductive. This phenomenon has been termed “The Hidden Cost of Reward” [1], “Corruption Effect” [2] and, more recently, “Cognitive Evaluation Theory” [3] or “Motivation Crowding Theory” [4].

This evidence has been largely neglected by economists. However, starting from the late Nineties, a growing number of empirical studies have coped with this puzzling phenomenon. This body of research substantially confirms the relevance of the Motivation Crowding Effect (MCE), both from laboratory experiments [5], [6] and field research [7]–[9].

Prompted by these empirical results, several studies have attempted to reconcile economic and psychological views, developing formal models that clarify the conditions under which the MCE may arise. This body of research extends and refines along two directions a basic strategic setting, in which contractual relationships are vitiated by potential conflict of interests arising from asymmetric information. Typically, this class of games consider a principal that contracts another party (agent) to perform some action; since the action is costly to the agent and his decision is costly to observe the agreement gives the agent the incentive to defects. The first approach [10], [11] considers an agency game with bilateral asymmetric information in which both the principal and the agent do not know something known by the other party which is relevant to their decision. For example, an employee (the principal) may know better than the worker (the agent) the toil and trouble required by the task; in this case the agent may infer from an explicit reward an excessive weariness, thus weakening his/her intrinsic motivation. If the reward is viewed as a strategic device that conveys information on some hidden, unpleasant, feature of the task the external incentive offered by the principal to the agent may reduce agent's effort and performance. The second approach assumes that the change in behaviour due to an external intervention does not reflect a change in the information set of the agent, but is attributed to a change in preference [12], [13]. Under this perspective, intrinsic motivation is modelled as an additional argument within a standard specification of agent's utility function. Since intrinsic motivation is assumed to adversely responds to explicit monetary reward, the resulting welfare loss may cause a lower effort and cooperation on the part of the agent.

Despite the different interpretations at the basis of the MCE, these two approaches share the implicit view that there is no room for intrinsic motivation to be a relevant aspect of observed behavior outside a strategic context. From this arise two important issues. On the one side this approach may determine the misleading view that the relevance of intrinsic motivations is restricted to a specific range of social situations. Actually, as pointed out by Aristotle more than 2,300 years ago, “man is by nature a social animal”, which, translated into the language of modern social sciences, exactly means that individuals are intrinsically motivated to social relations. It follows that whatever social interaction may be sustained by a motivation, related to the value of social interaction by itself, and distinct from the explicit goal that actually prompts the behaviour. Not only this implies that the bias of external rewards may arise in social situations not involving explicit perspective-taking activity, but also that outside explicit social contexts the role of motivations, and their potential conflict with external incentives, shouldn't arise as a relevant phenomenon. On the other side, the MCE should necessarily be the consequence of cognitively controlled processes undertaken by the agent. This issue has never been of any empirical concern by the above literature. It follows that it remains unclear, whether the implicit assumption that the MCE results from a conscious mental process is grounded on some kind of evidence or whether it is the consequence of the effort to formalize the MCE within a theoretical framework that retains the basic assumption of rationality.

Actually, we think that this question has never been considered by the above literature, mainly as a consequence of the scarce interaction between research undertaken within different social science fields. Indeed, a vast body of research in social psychology has demonstrated the importance of uncontrolled processes in shaping individuals' behavior [14]–[16] and, more recently, the debate has focused on the importance of motivation in unconscious processes [17]–[21]. However, this body of research is mainly concerned with the effectiveness and appropriateness of action in response to automatic evaluation, mainly to show how the unconscious provides individuals with effortless decision devices able to effectively purse a given goal both in individual contexts [22], [23] and in social contexts [24], [25]. On the contrary, our interdisciplinary perspective motivates a slightly different design, in the sense that we investigated under what conditions changes in external incentives may interact with social motivations to determine different patterns of behaviour, outcomes of an unconscious processing.

With regards to these considerations, the focus of our experiment was to verify whether different ways to distribute a given amount of money, affects effort and cooperation within a context where interaction between individuals does not involve any explicit process related to emotional cues and/or to strategic or “perspective-taking” considerations. Furthermore, since we wanted to investigate if different rewarding schemes influence behavior at a very low level, we avoid external incentives strictly contingent on performance by modulating a fixed amount of money within different experimental conditions, and focusing the attention on behavior variations revealed through activity changes in the muscle mainly involved during the execution of a motor task. To this purpose, twelve pairs of participants, prevented from any visual or verbal exchange, were submitted to a simple motor coordination task. Each couple had to cooperatively hold a small sphere between their right index fingers and to drop it alternately into one of two containers placed below their hands, while electromyography of the right *first dorsal interosseus* (FDI) muscle of each participant was recorded. Each successful trial was differently rewarded with a given amount of money according to the experimental condition, and the rewarding rules were communicated before starting each session. Consequently, for the same action (e.g., pushing the sphere into the left-side container) each participant could receive a reward in one session but not in another. The total monetary reward gained by each subject in each condition was always the same. Finally, we correlated muscle involvement with the scores obtained in a social attitude questionnaire to verify if the stock of social capital covertly modulates motor behavior.

## Methods

### Subjects

Twenty-four female participants were recruited among students of the Law Department of the University of Ferrara (mean age 26+/−3). All of them were naïve to the purpose of the experiment, were right-handed according to the Oldfield questionnaire [26] and gave their informed consent. They were divided into two subgroups (the “Green” and the “Yellow” group) of 12 participants, and kept in separate rooms after their arrival at the lab. Twelve pairs of subjects were then formed by extracting randomly one partner from each subgroup. Each pair, composed by one Green and one Yellow subject, was submitted to an experimental session lasting approximately 30 minutes.

### Questionnaire

In the first stage of the experiment the subjects were asked to answer a written questionnaire based on the Social Capital Community Benchmark Survey (SCCBS) [27].

Following the SCCBS we employed the answers provided by subjects to build several indexes aimed at measuring individual stock of social capital (see Appendix S2 for details).

### Coordination game

Before entering the lab room, subjects have been invited to remove rings, bracelets, nail enamel, or other kind of decoration, that could have made them recognizable by the other subjects. At the beginning of the experiment, two subjects entered the experimental room from two different doors, standing one in front of the other, their face and trunk hidden by a curtain. Thus, during the experimental session subjects never saw each other. Moreover, they were strictly recommended not to speak to exclude any possible recognition based on subject's voice.

Subjects were requested to pose their forearms on a Plexiglas surface with a square hole in correspondence of their hands. Twenty centimeters below the Plexiglas was set an apparatus constituted by two adjacent containers of equal size, with the partition side aligned with participants' sagittal plane. At the beginning of each trial a small glass sphere (1 cm diameter) was placed between the extended right index fingers of the two subjects, and subjects were requested to stay on this position (starting position) until the go-signal. In this position the sphere was exactly above the border between the two containers placed 20 centimeters below subjects' hands. Subjects' index fingers were dressed with a soft sponge to avoid finger flexion during the game, and to increase the attrition surface to better keep the sphere in the proper position.

**Figure 1:**
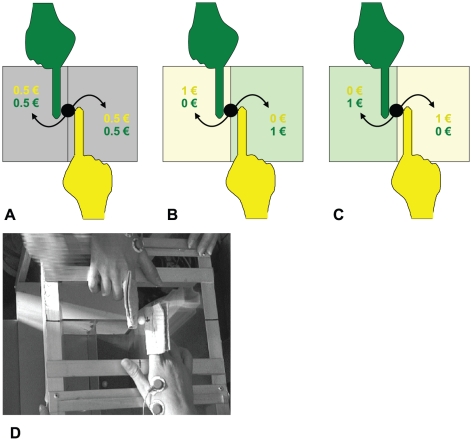
The experimental apparatus used in the three experimental conditions. Subjects' hands laid on a Plexiglas plate (see D) with the two index fingers positioned in correspondence of a square hole (the rectangle shown in the figure). Under the Plexiglas plate, at a distance of 10 cm from it, there were two containers (the two grey areas shown in panel A) where the subjects had to drop the sphere held by their index fingers according to the specific instructions provided for each experimental conditions. The moment at which the sphere touched the floor of the container was detected by a load cell. The monetary incentives associated to the three experimental conditions were the following: Condition 1 (A): each subject (Yellow and Green) get € 0.50 at any trial. Condition 2: the Yellow (Green) subject is coupled with the Yellow (Green) container; the pushing subject gets € 1 while the pulling one gets zero. Condition 3: the container are reversed; the pushing subject gets zero and the pulling one gets € 1.

Each pair of subjects was asked to play 30 trials of a simple motor ability game. The 30 trials were subdivided into three experimental conditions (*C*_1_, *C*_2_ and *C*_3_) of ten trials each, blocked into three experimental sessions, the presentation of which was pseudo-randomly balanced across pairs. At every trial subjects followed the instruction given by the experimenter indicating to drop the sphere alternately into the two containers. The difference among conditions *C*_1_, *C*_2_ and *C*_3_. consisted in the monetary incentive associated to each trial successfully performed by subjects. Specifically, in *C*_1_, putting the sphere into either target container yielded a reward of € 0.50 to each subject (Figure 1). In *C*_2_ and *C*_3_, two colored sheets, one green and one yellow, were placed onto the floor of each container, defining the Green and the Yellow container. The allocation of rewards coupled containers and subjects of the same color. When the sphere was successfully dropped into the target container a € 1 reward was received by the correspondent colored subject only. In *C*_2_, the Green (Yellow) container was placed at the left side of the Green (Yellow) subject: the winning subject had to execute an index finger abduction (contraction of the FDI muscle) to push the sphere towards the container (Figure 1B). In *C*_3_, the colors of containers were reversed, so that the Green (Yellow) container was placed at the right side of the Green (Yellow) subject: the winning subject had to execute an index finger adduction (FDI muscle not involved) to “pull” the sphere towards the container (Figure 1C). The total money reward gained by each subject was € 5 in each condition (€ 15 total).

### EMG Recordings

Electromyographic potentials (EMG) were recorded from right *first dorsal interosseus* (FDI) muscle by using Ag-AgCl surface electrodes (diameter 6 mm) glued to the subjects' skin according to a tendon-belly configuration. After online rectification and integration (time constant 50 ms) EMG signal was continuously recorded during the experiment and fed to a personal computer for the successive analysis. A custom-made software acquired the two filtered EMG at 25 Hz, a frequency fast enough to correctly sample the integrated - i.e. smoothed - signals. The instant at which the ball touched the bottom of the target container was detected by means of a load cell supporting the container itself. The signal from the load cell, appropriately amplified, was continuously acquired during the experiment by the same acquisition software used for EMG recordings and at the same sampling frequency.

### Data Analysis: ANOVA

For each trial, ten EMG samples, acquired from the pushing subject and concerning the 400 ms before the fall of the sphere into the container, were averaged and considered for the analysis. The averaged data from each subject, acquired during the three experimental conditions, were then normalized (z-score) and submitted to a one-way analysis of variance (ANOVA). The considered factor was Experimental Condition, a three levels, within-subjects, factor. Post-hoc analysis (Newman-Keuls, p<0.05) was then performed to verify the significant differences between individual conditions.

### Data Analysis: Regression Model

Our data set is distributed along four relevant dimensions: time, trials, subjects and conditions. In particular, since each trial has a different number of observations (i.e a different time length), to perform a regression analysis we had to balance our panel data set. To this purpose, we synchronized all trials with respect to the EMG peak of the pushing subject (i.e the instant at which the sphere was released, starting to drop into the container) and kept 12 observations before this point in time. This allowed us to construct a balanced panel data set of a total of 4,320 observations.

The potential information of our multi dimensional stock of data is not fully exploited by standard analysis of variance, since ANOVA does not control for many potential sources of variability, such as the muscle effort exerted by subject's couplemate, or individual fixed effects. Therefore, we considered the following dynamic multiple regression model:
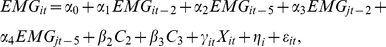
The dependent variable 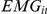 is subject *i*'s EMG signal at time *t*, when involved in pushing the sphere towards the target container. The right-hand side of the equation models the set of explanatory variables. Specifically, 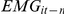 is the lagged EMG of subject *i* and 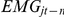 (*n* = 2,5) is the lagged EMG of subject *j* (couplemate of subject *i*). Lags have been set at 2 and 5 time periods (n = 2, 5). This accounts for a period of time ranging from 80 ms (2 * 40 ms, being the sampling frequency 25 Hz) to 200 ms (5 * 40 ms). This choice was based on the observation that when a perturbation is applied during a precision grip a latency of 60–80 ms is required to increase the grip force to restore an adequate safety margin, preventing frictional slips [28]. Thus, we defined this time range in order to include the minimal reaction time to a change in the load force applied by subject *j*, plus a possible delay determined by the fact that the grasping requires a coordination between two subjects and not only between two fingers of the same hand. To perform successfully the task it is required a continuous exchange of information between subjects, by the pressure exerted by their index fingers. The 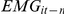 variables reflect the intention of subject *i* to push the sphere into the target container. At the same time, since the task requires the collaboration of subject *j*, the lagged 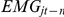 take into account that subject *i*'s effort depends on the opposition force exerted by subject's *j* finger. Thus, the dynamic part of the regression model represents the motor communication between subjects *i* and *j*. Other factors that might have influenced the motor behavior of subjects could have been determined by strain or stress and learning-by-doing. To account for these factors, we introduced in vector 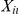 the time length of trials and the sequence order of trials over the entire experiment. The reason of our choice is that lengthy trials may have been more expensive in terms of attention, thus affecting the effort spent by subjects. Furthermore, subjects' effort might have been differently modulated over the course of the experiment, due to a better knowledge of her couplemate and/or to the improvement in their motor ability. Several non observable characters of subjects (such that religion, education, family conditions etc..) may influence the dependent variable. The term 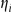 represents a vector of individual dummies, that control the regression model for this individuals' heterogeneity. Finally, 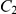 and 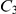 are two dummies for condition 2 and 3 respectively, controlling for experimental conditions instructions.

## Results

### Behavior and Electromyography

As shown in Table 1 subjects were able to coordinate almost perfectly in all three experimental conditions, with only a negligible proportion of inefficient outcomes (2.7% of total observations), uniformly distributed across conditions.

**Table 1:** Outcomes of the game for each condition.

*Condition*	*Green wins*	*Yellow wins*	*Inefficient outcomes*	*Total (12 pairs×10 trials)*
1	58	58	4	120
2	59	58	3	120
3	59	58	3	120
*Total*	176	174	10	360

**Figure 2:**
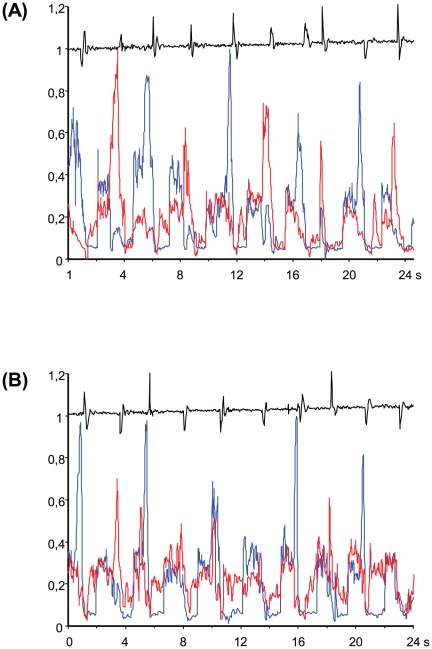
Typical *first dorsal interosseus* electromyographic signal rectified, integrated (time constant, 0.05 s) and intra-subject normalized (z-scores), as recorded from two subjects (red and blue traces) during the interaction game. Panel A, Condition 1; panel B, Condition 3. The signal recorded from the load cell is shown in black and indicates the ten times the glass sphere fell into the container, signaling the end of each trial. The figure depicts ten subsequent trials (sampling frequency, 25 Hz). Abscissas, seconds; ordinates, arbitrary normalization units (see text).

Figure 2 depicts the typical EMG traces recorded from both subjects' FDI muscles (blue and red traces) and the signal recorded from the load cell, detecting the instant at which the sphere, after its releasing, touches the floor of the container (black trace), during condition 1 (A) and 3 (B).

As it appears from Figure 2, at the beginning of each trial there is an increase of both subjects' EMG determined by the involvement of subjects' index fingers in maintaining the glass sphere in the starting position. After the go-signal (not indicated in the figure), one of the two subjects starts to exert a phasic effort to push the sphere into the assigned container, as revealed by a clear peak, slightly anticipating the load cell signal. While in panel A the blue and the red peaks clearly alternate, in panel B the trend is less clear, showing some degree of superimposition of the two traces during some of the trials. Note that in both conditions the instructions were exactly the same: “Place the sphere into the target container”. The only difference between the two conditions concerned the monetary reward. In Condition 1, each member of the pair was winning at any trial, while in Condition 3, each member of the pair was winning only when the target container was the one at her right side, requiring the pulling of the sphere towards the container requiring an index finger adduction (FDI muscle not involved).

**Figure 3:**
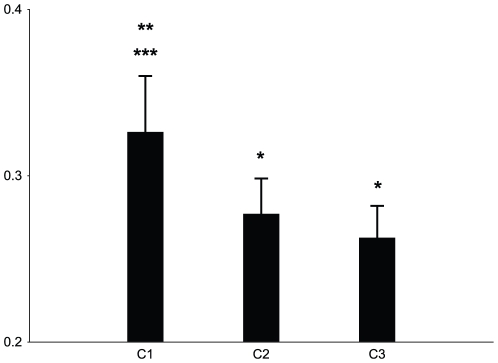
Mean values of EMG signals recorded from the FDI muscle for all subjects in the three experimental conditions, when pushing the sphere into the target container placed at her left side. Whiskers above each histogram depict the standard error of mean. Ordinates: *z*-score of EMG signals. Asterisks indicate the presence of a significant difference between conditions (*, difference from Condition 1 (C1); **,*** difference from Conditions 2 (C2) and 3 (C3), respectively).

This qualitative difference between conditions is quantitatively shown in Figure 3, depicting the average values of FDI muscle EMG, recorded from each subject while pushing the sphere into the target container placed at her left side in the three experimental conditions. EMG data, after normalization, were averaged subject by subject (N = 24) by pooling the last 12 trials before the signal of the load cell signaled the fall of the sphere.

ANOVA performed on the normalized data with Experimental Condition as three levels within-subjects factor (see Figure 2) showed that Experimental Condition was statistically significant (F(2,46) = 4.48, *p* = 0.017). Post-hoc analysis (Newman-Keuls) revealed that EMG activity of *C*_1_ was significantly (*p*<0.05) stronger than that of *C*_2_ and *C*_3_. However, as indicated in Table 1, the game outcome does not reflect this difference, and subjects, interviewed at the end of the experiment, never reported the voluntary use of different strategies in the different conditions.

### Questionnaire

One of the aims of the present work was to verify if different levels of social capital modulates muscle involvement of the pushing subjects, in response to different monetary incentives among conditions. Using the questionnaire's answers, we built up three indicators (*SC1*, *SC2*, *SC3*) to sort subjects according to their attitude to coordinate and cooperate for mutual benefit (see Appendix S2 for details). For each of these indicators subjects have been divided into two subgroups with respect to the index-related median score, defining the high- (*H* = above median) and the low (*L* = below median) Social Capital groups of subjects.

### Regression Results

The relevant estimation results are presented in table (2) below. The first column (POOL) reports the estimation results for the entire set of subjects (24). The other six columns provide results relative to each high/low prosocial sub-groups according to indicators SC1, SC2 and SC3. In particular, HSC_z_ and LSC_z_ (z = 1, 2, 3) refer to High and Low prosocial individuals, respectively.

Even with the rich specification of explanatory variables inside the regression model, all simultaneously engaged to account for the variability of the effort recorded from the pushing subjects, it still emerges that on average subjects exerted a lower pushing effort in condition 3 than in condition 1: only the estimated coefficient of 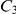 is negative (−0.0244) and 5% significant (*t* = 2.44). However, once we distinguish between high social capital and low social capital subjects, the estimated coefficients of 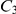 is negative and significant at a 1% level in the low-prosocial sub-sample, only. This pattern arises whatever index of social capital is used. Moreover, the coefficient on the dummy 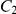 turns out not significant in all regressions, indicating that no difference in effort is detected between Condition 1 and Condition 2. Despite the ANOVA reported that subjects significantly spent a lower effort in Condition 2 than in Condition1, in the light of Table 2 this result appears spurious. Indeed, the regression tells that the EMG difference between conditions 1 and 2 does not reflect any change in external incentives schemes, but more likely the variability in the other set of explanatory variables.

**Table 2:** Ordinary Least Squares Regression keeping 12 observations before the maximum EMG level, included.

	POOL	HSC_1_	LSC_1_	HSC_2_	LSC_2_	HSC_3_	LSC_3_
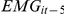	0.1121(4.59)***	0.1439(4.22)***	0.0838(2.32)**	0.1840(5.78)***	0.0567(1.56)	0.1560(4.72)***	0.0924(2.46)**
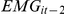	0.2898(12.69)***	0.2918(8.49)***	0.2634(8.78)***	0.2634(7.67)***	0.2724(8.76)***	0.2294(6.73)***	0.3233(10.54)***
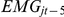	0.0898(3.82)***	0.0405(1.38)	0.1421(3.79)***	0.0473(1.64)	0.1552(4.06)***	0.0615(2.11)**	0.1196(3.07)***
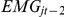	0.0330(1.41)	0.1153(3.61)***	−0.0451(1.38)	0.0748(2.46)**	−0.0073(0.21)	0.0474(1.61)	0.0281(0.75)
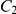	−0.0041(0.40)	−0.0095(0.70)	0.0003(0.02)	−0.0098(0.72)	0.0040(0.27)	−0.0079(0.59)	0.0015(0.10)
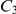	−0.0244(2.44)**	0.0156(1.10)	−0.0630(4.49)***	0.0105(0.73)	−0.0535(3.82)***	−0.0093(0.67)	−0.0389(2.72)***
Constant	0.1044(4.46)***	0.0867(2.66)***	0.1322(0.3.89)***	0.1319(3.71)***	0.1092(2.80)***	0.1748(4.56)***	0.0632(2.05)**
Observations	1755	855	870	885	870	880	875
R-squared	0.3955	0.4774	0.3455	0.4276	0.4121	0.4472	0.3665

Robust t statistics in parentheses:

*significant at 10%;

**significant at 5%;

***significant at 1%.

Normalization over the entire data set.

Looking at the dynamic component of the regression, coefficients of lagged variables are positive and significant, suggesting that each couple of subjects successfully tried to coordinate their index fingers as a pair of agonists. However, considering the magnitude of coefficients for different groups of subjects substantial differences emerge between high social capital (High SC) and low social capital (Low SC) individuals. In particular, the following two results appear to be relevant (formal tests are provided in Appendix S1 )

R1) The coefficients of the dynamic part of the regression (autoregressive component) decrease with farther time lags, both in the High SC and Low SC groups

R2) Result 3 emerges looking at the coefficients describing how subjects' current effort depends on the past effort of her couplemate. In the High SC group the coefficient at lag (−2) is greater than the corresponding coefficient at lag (−5) and the reverse patterns occurs within the Low SC group. Moreover, it appears that coefficient at lag (−2) is higher in the High SC group than in the Low SC group, while the reverse pattern is observed at lag (−5).

For both high and low social capital subjects the autoregressive component of the regression model (the lagged 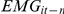 variables) shows that the current effort 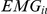 of subject *i* is positively linked to her own past efforts, and that the magnitude of the coefficients decreases the farther-off are the lags (result R1). This is consistent with Figure 2, which shows that intensity of muscles effort progressively increases, and reaches its peak at the instant at which the sphere is dropped.

Result R2 describes how the current reaction of subject *i* depends on past motor behavior of subject *j*. Overall, estimated coefficients are significantly non negative. However, looking at the size of coefficients it emerges a striking difference between high and low social capital individuals. Current muscle effort of high social capital subjects is influenced mainly by the more recent behavior of their couplemates, while current effort of low social capital subjects is better explained by the more distant behavior of their couplemates. Considering high social capital subjects, the estimated coefficients on 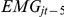 are not significantly different from zero in two of the three regressions (HSC_1_ and HSC_2_) and significant at the 5% level but close to zero in the HSC_3_ case. On the contrary, coefficients on 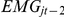 are positive and significant in HSC_1_ and HSC_2_ and not significant in HSC_3_. Exactly the reverse pattern occurs with low social capital subjects: coefficients on 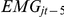 are significant at a 1% level, while those on 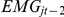 are not significant in all cases (LSC_1_, LSC_2_ and LSC_3_). This evidence shows that, compared to high social capital subjects, low social capital participants exhibited a delayed response to stimuli coming from changes in effort in subject's opposing finger. This may suggest that high social capital individuals might have been prompted by a stronger intrinsic motivation, which resulted in a more effective motor coordination.

## Discussion

An impressive body of social and cognitive psychology studies reports evidence supporting the view that external rewards, typically monetary ones, undermine intrinsic motivation (see [3] and [4] for an extensive survey and meta-analysis). These findings contradict the behaviour predicted on the basis of the standard selfish-rationality assumption, which is at the core of economic reasoning. As a consequence, since the late 90 *s* an increasing number of experimental, empirical and theoretical studies have explored this puzzling issue. This body of research shares the view that the proper frame in which to consider this phenomenon is a principal-agent game context (see [4] for a discussion of the major economic studies identifying crowding effects). This approach represents a fruitful context to investigate the interaction between extrinsic and intrinsic motivations. However, it is not the proper setting to explore if the side effects of external incentives on intrinsic motivations might arise as an automatic process, since, due to the strategic environment, the crowding out/in of intrinsic motivations necessarily follows from an explicit perspective-taking activity undertaken by subjects.

In the light of these considerations, we set up an experimental framework devoid of any complex perspective-taking activity, aimed at investigating whether the modulation of a given monetary reward affects effort and performance of participants. Pairs of subjects, prevented from any visual or verbal interaction, were engaged in a pure motor coordination game divided into three experimental conditions, perfectly identical from the point of view of the required motor task. Moreover, the monetary stake associated to each condition was exactly the same. Each couple of subjects was asked to hold a small sphere between their right index fingers and to alternately drop it into one of two containers placed below their hands, while electromyography of participants' right FDI muscle was recorded. This muscle has the function to abduct the index finger away from the middle finger. Thus, it is the muscle more involved in pushing the sphere towards the leftmost container, while it remains relaxed when the participant is asked to place the sphere into the rightmost container by exerting a finger adduction. Our aim was to compare FDI muscle activity when participants were asked to push the sphere into the leftmost container under different rewarding schemes. In *C*ondition 1 the completion of each trial entailed an equal reward assigned to both (pushing and opposing) subjects. In *C*ondition 2 at each trial only the subject who had pushed the sphere towards the leftmost container obtained the reward. Therefore, in Condition 1 and Condition 2 FDI muscle involvement in pushing the sphere was coupled with a monetary reward. On the contrary, in Condition 3 the reward was assigned to the opposing subject only. Thus, in all trials, FDI muscle involvement in pushing the sphere was not associated with any specific monetary reward. It should be stressed that, since the total monetary reward allocated to both subjects upon completion of the sequence of trials did not change across conditions, the overall *external motivation*, that prompted the motor performance was the same in each experimental condition. Since subjects were able to coordinate almost perfectly their movements across conditions 1, 2 and 3, their behavior is consistent with the conscious perceiving of this external incentive. Despite from a distributional point of view it does not emerge any difference in behavior associated with alternative incentive protocols, substantial differences arise from EMG data processing, revealing that not only muscle involvement in executing the same motor act is affected by different allocations of an identical monetary reward, but also that the modulation of the effort is correlated with the degree of prosocial propensity of subjects. To measure the social attitude of participants we used the answers to the questionnaire taken from Putnam's Social Capital Benchmark Survey to construct three indexes of social capital, that we used to split the sample of subjects into high and low social capital individuals. With respect to these two groups of individuals our main result is that high-prosocial subjects performed the task without any significant difference among conditions, while low-prosocial subjects exerted a significant lower effort in Condition 3 than in *C*ondition 1.

When a small object is gripped between the tips of the index finger and thumb and held stationary in space, the applied grip force is synchronically balanced to optimize the motor behaviour. In addition, the control of the grip force is automatically influenced by the weight of the object (load force) and by a safety margin factor related to the individual subject [29], [30]. Since this is fundamental to avoid the accidental drop of the object, within the context of our experiment, the level of safety margin set by subjects to avoid errors must be related to the *intrinsic motivations* that sustained action toward the desired goal. These considerations highlight the baseline for discussing our results. Specifically, we consider that the two index fingers of pair of subjects acted as pairs of agonists, and that statistically significative changes in effort detected through the EMG recording relates to the intensity intrinsic motivations. We assumed that the overall external motivation was the same in all three experimental conditions, because each participant received, the same reward in all conditions and the motor task was performed following the instructions of the experimenters, to avoid any perspective-taking activity. Moreover, upon asking participants at the end of the experiment, none of them affirmed to have consciously changed her effort or strategy across different conditions. In the light of these considerations, it is conceivable that the modulation of the effort in response to different rewarding schemes was the consequence of an automatic and unconscious mental process. Therefore, the MCE of intrinsic motivations due to external incentives, that ultimately determined the level of application and diligence exerted by subjects (the safety margin factor), may arise as an unconscious outcome outside a strategic context, even in response to “weak” external incentives changes, such as a slightly different way to deliver a given amount of money.

In Condition 1 the completion of each trial entailed an equal reward assigned to both subjects. In this respect, the surplus resulting from the cooperation was equally divided between subjects. Following an economic terminology, in condition 1 the benefit of cooperation was not excludable, in the sense that no individual could be excluded from enjoying a slice of the surplus generated by the coordinated efforts. In Condition 2 and 3 cooperation is still productive, but within trials only one participant was rewarded. This character of excludability in Conditions 2 and 3 introduced a substantial difference with respect to Condition 1: reciprocity. In social psychology (as well as in game theory) reciprocity means that people reward kind action and punish unkind ones. In the present context reciprocity has sustained in Conditions 2 and 3 an implicit agreement between subjects, in the sense that results in Table 1 are consistent with the statement: “I help you to win €1 if you help me to win €1”. However, the excludability of surplus between Conditions 2 and 3 is asymmetrical, since in Condition 2 it is the pushing subject that it is rewarded, while in Condition 3 it is the opposing subject. Actually, this asymmetry introduced roles within trials. Specifically, looking at the mechanics of the coordination, it is fairly clear that if the opponent's finger started moving before the other one started pushing, the sphere would have been fallen in the wrong container. Since the event of accidental drop of the sphere has occurred in a negligible proportion, we can safely claim that the subject opposite to the pushing one as not started to move before the pushing subjects had started to push. Since, at each trial it was the pushing subject that decided to start action, while the opposing one waited for her couplemate's decision, following the metaphors of the game theory we attribute the role of leader to the pushing subject and the role of follower to the opposing subject. Following the metaphor outlined above, we can interpret our result in the light that Conditions 1 and 2 share the feature that the leader is rewarded, while Conditions 2 and 3 share the characteristic that cooperation is sustained by reciprocity.

Our main results are that high social capital participants subjects exhibited no significant reaction to the modulation of external reward within conditions. In this respect, the intrinsic motivations sustaining cooperation was not affected by removing the monetary incentive of the leader (the pushing subject) in Condition 3. In this sense, high social capital participants displayed strong reciprocity, which caused them to be insensitive to changes in external motivations. On the contrary, low social capital participants exerted a significative lower effort in Condition 3 than in Condition 1, but no difference in effort is detected between Conditions 1 and 2. Following our line of interpretation, this sample of subjects actually perceived a zero monetary incentive as they played as leader in condition 3, causing a lower effort spent in the task. In this respect, they actually responded to extrinsic motivation consistently with a selfish-rationality argument. However, effort spent in condition 1 does not significantly differ from effort recorded in condition 2. Thus, it seems that the non excludable character of surplus in condition 1 did matter. In condition 1 whatever container did the sphere have been dropped subjects were rewarded, thus reciprocity was not relevant. In this sense, this group of this group of subjects exhibited only weak reciprocity, since they showed some degree of aversion to reciprocate unless they were externally rewarded.

In terms of motivational literature, the above discussion suggests two final considerations. First, reciprocity appears a relevant dimension of intrinsic motivations in social interaction, and, more interestingly, the propensity to reciprocate depends on the stock of social capital. Since the accumulation of social capital can be an explicit policy target on the part of public institutions, our results suggest a precise channel, micro-founded on the behaviour of the single individual, through which investment in social capital might display their effects. More specifically, only when individuals are poorly endowed with social capital social interaction via market-transactions (i.e. through external motivations incentives) is effective. On the contrary, when individuals are integrated by high levels of social capital, their behavior may react to changes in relative prices in opposition to what is expected on the basis of a standard economic argument. Second and more importantly, the effects of extrinsic rewards on intrinsic motivation does not rely upon any explicit cost-benefit evaluation, stemming from a controlled cognitive process, but may result automatically as an unconscious outcome. This may reflect the specific monetary character of the external motivation. It is a well established result that several external stimuli may “prime” subjects, conditioning in an uncontrolled way their behaviour [31], [32], [20]. A more recent study, however, has showed the precise behaviour's bias due to the priming of money [33], which supports the interpretation of our results. The main result of this study shows that “money brings about a self-orientation, in which people prefer to be free of dependency and dependents“ (p. 1154). In this respect the “aversion for reciprocity” argument we used in the discussion before may just be reversed by using a notion of “preference for the self-supporting”, which is exactly the consequence of the priming of money according to Vohs et al. [33]. On the one side, our results find additional supports from the subliminal effects of money investigated in this study, on the other side they refine this evidence since we show that the “priming” effect of money is modulated by the social relevant experiences of individuals (social capital).

## Supporting Information

Appendix S1 (DOC)

Appendix S2 (DOC)
