# Supplementary material for: Electromyographic Activity of Hand Muscles in a Motor Coordination Game: Effect of Incentive Scheme and Its Relation with Social Capital
Source: PLoS One. 2011 Mar 25;6(3):e17372. doi: 10.1371/journal.pone.0017372 (PMC3064577; doi:10.1371/journal.pone.0017372)
Supplement: Appendix S1 — (DOC) [file pone.0017372.s001.doc]

Appendix S1

*Formal testing for results R1, and R2.*

In ‘within’ regression comparisons we tested the Null Hypothesis of equality between coefficients by using a generalized F-statistics, with (1, *N*-*K*) degrees of freedom, where *N* is the sample size and *K* the number of regressors. When comparing coefficients estimated from two regressions (High-Social capital and Low Social Capital) we used the test proposed by Brame et al [1], which follows a t-distribution with (*N*-4) degrees of freedom. Also in this case the null hypothesis assumes that the difference between coefficients is zero. Results are reported in tables below, where $\alpha_{1},\alpha_{2},\alpha_{3},\alpha_{4}$refers to the coefficients of regression presented in text, *H* and *L* refers to High social capital and Low social capital subjects, and (SC1, SC2 , SC3) refers to the three indices of social capital employed in the regression analysis. In the tables below are reported the computed statistics (the Null Hypothesis of equality between coefficients is rejected at the *10%, **5%, ***1% significance level) and the direction of the difference (+ positive, - negative)

Result 1 (R1)

|  | $\alpha_{1}^{H}-\alpha_{2}^{H}$ | $\alpha_{1}^{L}-\alpha_{2}^{L}$ |
| --- | --- | --- |
| *SC1* | + (6.89)*** | + (11.56)*** |
| *SC2* | + (2.20)* | + (15.52)*** |
| *SC3* | + (1.89) | + (17.66)*** |

Result 2 (R2)

|  | $\alpha_{3}^{H}-\alpha_{4}^{H}$ | $\alpha_{3}^{L}-\alpha_{4}^{L}$ | $\alpha_{3}^{H}-\alpha_{3}^{L}$ | $\alpha_{4}^{H}-\alpha_{4}^{L}$ |
| --- | --- | --- | --- | --- |
| *SC1* | + (2.62) * | - (9.77)*** | + (3.47)*** | - (2.13)*** |
| *SC2* | + (0.37) | - (6.73)*** | + (1.,77)** | - (2.26)*** |
| *SC3* | - (0.10) | - (1.94)* | + (0.42) | - (1.18) |

Reference:

1. Brame R, Mazerolle P, Piquero A (1998) Using the corrects statistical test for equality of regression coefficients, Criminology 36:859-866.
