# Supplementary material for: Electromyographic Activity of Hand Muscles in a Motor Coordination Game: Effect of Incentive Scheme and Its Relation with Social Capital
Source: PLoS One. 2011 Mar 25;6(3):e17372. doi: 10.1371/journal.pone.0017372 (PMC3064577; doi:10.1371/journal.pone.0017372)
Supplement: Appendix S2 — (DOC) [file pone.0017372.s002.doc]

Appendix S2

*The Questionnaire and the social capital indexes*

Subjects where asked to answer a questionnaire designed to follow very closely the SCCBS [27]. An increasing number of applications, from sociology to health economics, political science, business management, human resources and politics have used the concept of Social Capital, depending on circumstances, as synonym of rather diverse concepts, such as “generalized trust'', “civic engagement”, “religious belief” or “group interaction”. Using the questionnaire’s answers we built up indicators of these characteristics to sort our subjects according to their attitude to coordinate and cooperate for mutual benefit.

Following the SCCBS we built up six indexes. Civic participation (cp) was constructed (see index CIVPART in [27]) as the average of three different questions, meant to measure individual involvement in civic and political activity, such as working for a political party in the past year (q1.2), attending political meetings in the past year (q1.5) and signing petitions in the past year (q1.7):

*cp*=(q1.2+q1.5+q1.7)/3.

We also built an alternative index cpext, by adding subject’s answer to a specific question, namely q5.4 which asked how important was politics in their personal life (answer ranked from 4=“very important” to 1=“not important at all”:

*cpext*=(q1.2+q1.5+q1.7+(q5.4-1)/3)/4.

Faith-based Social Capital (fbsc) was an indicator (see index FAITHBAS in [27]) constructed as the average of two questions, designed to measure participation in the life of the local religious community such as going to church in the past week (q2.8), or going to church social function in the past month (q3.6)

*fbsc*=(q2.8+q3.6)/2.

By analogy with cpext, we also considered the following:

*fbscext*=(q2.8+q3.6+(q5.6-1)/3)/3.

Organized Group Interactions (ogi) was built (see index ORGINTER in [27]) as the average of six questions, designed to measure participation in the life of the local community such as serving as an officer of some club organization in the past year (q1.1), or in a committee for some local organization in the past year (q1.2), attending a public meeting of club or civic organization in the past month (q3.7):

*ogi*= (q1.1+q1.3+q1.4+q3.7)/4.

Informal Group interaction (igi) was an indicator (see index SCHMOOZ in [27]) constructed as the average of six questions, designed to measure participation in the informal social network such as having friends in for the evening in the past week (q2.3); going to the home of friends in the past week (q2.4); going to club, disco, bar or place of entertainment in the past week q2.11); going to friends’ house for dinner or evening in the past month (q3.4); having friends in for dinner or evening in the past month (q3.5); going to night club, disco, bar in the past month (q3.9):

*isi*=(q2.3+q2.4+q2.11+q3.4+q3.5+q3.9)/6

SCCBS also considers five additional indexes, based on social trust (STRSTCAT), group involvement without church participation (GRPINCAT), group involvement with church participation(GRP2CAT), diversity of friendship network (DIVRCAT), and composite racial group trust (RACETCAT). Due to an almost null variability in subjects’ answers (probably due to a higher homogeneity of our subject pool with respect to the relevant dimensions) we could not make any use of these additional indexes.

Finally, to rank subjects according to a composite scale of the relevant characteristics determining the attitude to coordinate and cooperate, we constructed three composite measures using the indexes above outlined:

*SC_1_*=(cpext+fbscext+ogi+isi)/4

*SC_2_*=(cpext+fbscext+ogi)/3

*SC_3_*=(cp+fbsc+ogi)/3.

*The Questionnaire*

Here we report the text (translated from Italian) of the questionnaire submitted to participants.

**Please answer to the following questions:**

**1) Which, if any, of these things have you done in the past year?**

- - 1.1 Served as an officer of some club or organization
  - 1.2 Worked for a political party
  - 1.3 Served on a committee for some local organization
  - 1.4 Attended a public meeting on town or school affairs
  - 1.5 Attended a political rally or speech
  - 1.6 Made a speech
  - 1.7 Signed a petition
  - 1.8 Wrote a letter to the paper
  - 1.9 Wrote an article for a magazine or newspaper

**2) Which, if any, of these things have you done in the past week?**

- - 2.1. Discussed politics
  - 2.2. Had dinner in a restaurant
  - 2.3 Had friends in for the evening
  - 2.4 Went to the home of friends
  - 2.5 Saw a movie
  - 2.6 Made a personal long distance call
  - 2.7 Read a book
  - 2.8 Went to church
  - 2.9 Watched a sports event on TV
  - 2.10 Went out to watch a sports event
  - 2.11 Went to club, disco, bar or place of entertainment
  - 2.12 Spent time on a hobby
  - 2.13 Wrote a personal letter or e-mail
  - 2.14 Received a personal letter or e-mail

**3) How many times, if any, did you do any of these activities in the past month?**

- - 3.1 Made a contribution to charity
  - 3.2 Did volunteer work
  - 3.3 Donated blood
  - 3.4 Went to friends’ house for dinner or evening
  - 3.5 Had friends in for dinner or evening
  - 3.6 Went to church social function
  - 3.7 Went to meeting of club or civic organization
  - 3.8 Went to dinner at restaurant
  - 3.9 Went to night club, disco, bar
  - 3.10 Went to live theater, opera, concerts
  - 3.11 Went to sporting event
  - 3.12 Went to the movies

**4) Which of the following things are part of "the good life" in your opinion?**

- - 4.1 A home you own
  - 4.2 A yard and lawn
  - 4.3 A second car
  - 4.4 A vacation home
  - 4.5 A swimming pool
  - 4.6 A happy marriage
  - 4.7 No children
  - 4.8 One or two children
  - 4.9 A job that pays more than average
  - 4.10 A job that is interesting
  - 4.11 A job that contributes to the welfare of society
  - 4.12 College education for my children
  - 4.13 Travel abroad
  - 4.14 A second color TV set
  - 4.15 Really nice clothes
  - 4.16 A lot of money

**5) For each of the following, indicate how important it is in your life. Would you say it is:**

1. Very important
2. Rather important
3. Not very important
4. Not at all important
5. I don’t know
   - 5.1 A home you own Family
   - 5.2 A yard and lawn Friends
   - 5.3 A second car Leisure time
   - 5.4 Politics
   - 5.5 Work
   - 5.6 Religion
   - 5.7 Service to others

**6) Taking all things together, would you say you are:**

4. Very happy

3. Quite happy

2. Not very happy

1. Not at all happy

0. Don't know

**7) With which of these two statements do you tend to agree with?**

**(CHOOSE ONE ANSWER ONLY)**

A. Regardless of what the qualities and faults of one's parents are, one must always love and respect them

B. One does not have the duty to respect and love parents who have not earned it by their behavior and attitudes

7.1 Tend to agree with statement A

7.2 Tend to agree with statement B

7.3 Don't know

**8) Generally speaking, would you say that most people can be trusted or that you need to be very careful in dealing with people?**

8.1 Most people can be trusted

8.2 Need to be very careful

8.3 Don't know

**9) Do you think most people would try to take advantage of you if they got a chance, or would they try to be fair?**

9.1 Would take advantage

9.2 Would try to be fair

9.3 Don’t know
